# Perception of action-outcomes is shaped by life-long and contextual expectations

**DOI:** 10.1038/s41598-019-41090-8

**Published:** 2019-03-26

**Authors:** Myrthel Dogge, Ruud Custers, Surya Gayet, Herbert Hoijtink, Henk Aarts

**Affiliations:** 1 Department of Psychology, Utrecht University, Utrecht, The Netherlands; 2 Donders Institute for Brain, Cognition and Behavior, Radboud University, Nijmegen, The Netherlands; 3 Department of Methodology and Statistics, Utrecht University, Utrecht, The Netherlands

## Abstract

The way humans perceive the outcomes of their actions is strongly colored by their expectations. These expectations can develop over different timescales and are not always complementary. The present work examines how long-term (structural) expectations – developed over a lifetime - and short-term (contextual) expectations jointly affect perception. In two studies, including a pre-registered replication, participants initiated the movement of an ambiguously rotating sphere by operating a rotary switch. In the absence of any learning, participants predominantly perceived the sphere to rotate in the same direction as their rotary action. This bias toward structural expectations was abolished (but not reversed) when participants were exposed to incompatible action-effect contingencies (e.g., clockwise actions causing counterclockwise percepts) during a preceding learning phase. Exposure to compatible action-effect contingencies, however, did not add to the existing structural bias. Together, these findings reveal that perception of action-outcomes results from the combined influence of both long-term and immediate expectations.

## Introduction

Perception is a crucial faculty for moving organisms. In order to support fast and efficient interaction with the environment, perceptual processing cannot merely rely on bottom-up sensory input, but instead requires top-down knowledge to shape and understand incoming signals^1^. As a consequence, perception is strongly affected by what one expects to perceive^2,3^. Perceptual expectations can be derived from a myriad of sources^3^. This includes not only the spatial and temporal regularities in the sensory signal itself, but also the actions of the observer (e.g., when turning a steering wheel, drivers tend to expect their car to turn in the same direction). In line with this idea, it has been proposed that action preparation is accompanied by the prediction of the sensory consequences of movement^4,5^. The neural computations involved in programming and comparing these predicted action-outcomes with actual input are thought to be an important signal for how humans experiences themselves as causal agents^6,7^. Hence, elucidating how motor expectations are formed and updated when interacting with a dynamic physical, and social, environment is crucial for understanding how the agentic sense of self emerges.

Expectations about upcoming events can develop across different timescales, and have been broadly divided into two categories^8^. Firstly, humans form sensory expectations by exposure to relatively stable statistical regularities in the environment across their lifetime. Such long-term (structural) expectations can for instance be observed in expert piano players, who are more likely to perceive an ambiguous tone pair as going up or down in pitch after pressing keys on a keyboard in a left-right order or right-left order, respectively^9^. Along similar lines, tones are generally judged as lasting longer when preceded by a movement of a longer duration^10^, the perceived number of visual events is biased by the number of preceding key presses^11^, and ambiguous motion percepts are perceived as moving in the same direction as accompanying bodily actions (a phenomenon known as action capture^12^). Secondly, perception is also affected by more temporary action-outcome expectations, that are more easily updated through contextual demands^8^. For instance, the perceived motion direction of ambiguous action-outcomes can be biased by acquiring new, arbitrary, associations between actions and following effects^13,14^.

In everyday life humans are regularly exposed to circumstances in which contextual expectations conflict with overlearned (structural) expectations. For instance, when reversing a car with a trailer into a parking space, turning the wheel has the opposite result to regular driving conditions (i.e., turning the wheel in a rightward direction will now result in a leftward turn). To date, little is understood about how fast expectations are updated in these kinds of circumstances, and more specifically, how long-term and short-term expectations jointly determine the predicted perceptual outcome of an action. Although a few studies have provided evidence for the notion that structural expectations can be modulated by contextual expectations^15,16^, such updating effects are not always observed^17^. In addition, action-based expectations have hitherto largely been ignored. One important exception is a recent study on the amenability of structural motion biases (i.e., the tendency to see ambiguous motion in line with one’s own movement^14^). However, whilst this study reports clear contextual learning effects, no consistent structural expectations were observed. This leaves open the question whether similar contextual updating would be observed in the face of more robust long-term expectations.

Another key issue that remains unclear from existing work is how expectations developed over different timescales persist over time. Specifically, existing studies do not address the question whether and how flexibly, structural expectations overtake contextual expectations after the latter expectations are no longer valid or reinforced by evidence.

Here, we present two studies in which we assessed the joint influence of structural and contextual expectations on the perception of action-outcomes, using ambiguous motion displays. In Study 1 participants manually operated a rotary switch, which initiated the presentation of a bistable rotating sphere that could be perceived as rotating in a clockwise or counterclockwise direction. In order to assess the interaction between structural and contextual expectations, a preceding learning phase was completed in which participants were exposed to action-motion contingencies that were either compatible (i.e., a clockwise movement resulting in a clockwise rotation of the sphere) or incompatible with structural expectations (i.e., a clockwise movement resulting in a counterclockwise rotation of the sphere). Finally, we included a baseline condition, in which test blocks were not preceded by a learning phase, thus allowing us to assess pre-existing (i.e., structural) action-effect anticipations.

To ensure that participants’ reported rotation directions genuinely reflected their perceived rotation direction (rather than a percept-unrelated response tendency), we devised a response-task that was orthogonal to our measure-of-interest: The sphere motion was divided into two successive epochs, and participants were asked to report whether or not a reversal of rotation direction had occurred. Because the rotation direction of the sphere in the second epoch was always unambiguous, we could infer the perceived rotation direction of the sphere in the first epoch (i.e., the initial percept) from the presence or absence of a reversal report, while at the same time alleviating a potential response bias.

Based on the research reviewed above, we firstly hypothesized to observe a general bias to perceive the initial rotation direction of the sphere in the same direction as one’s movement (i.e., following structural expectations). On top of that we expected a tendency to perceive the sphere to rotate in the most recently learned direction (i.e., contextual expectations). Finally, we expected the strength of this contextual bias to weaken as a function of time due to extinction of the learned contingencies. In Study 2 we conducted a pre-registered replication of Study 1.

## Results

### Study 1

#### Perceptual selection

**Figure 1:**
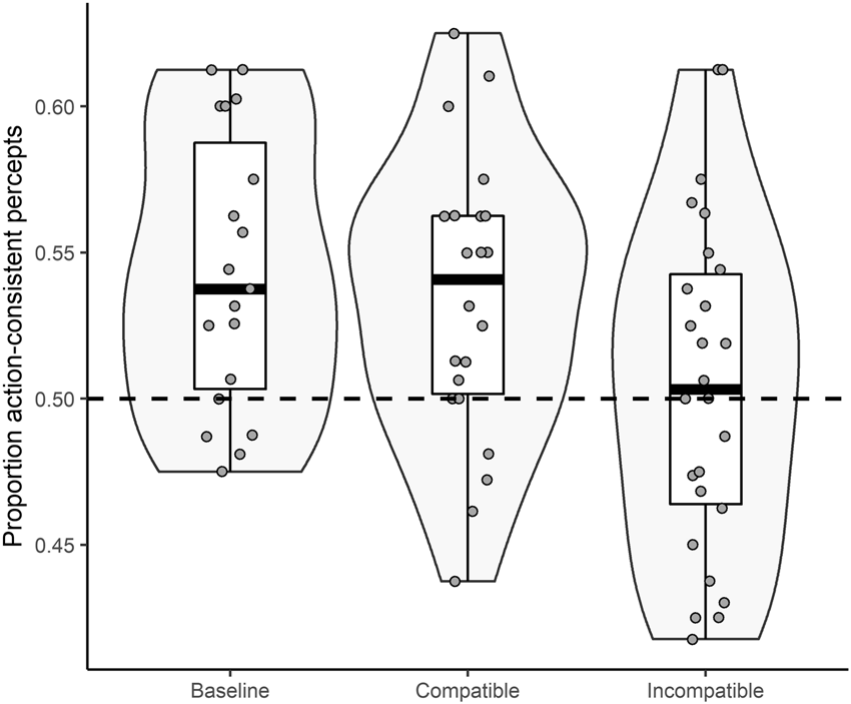
Proportion of action-consistent percepts for all three conditions in Study 1. Lower and upper box limit represent the 25^th^ and 75^th^  percentile respectively. Whiskers extend to the most extreme values that fall within 1.5 times the interquartile distance from the hinges of the box. Central lines represent the median. Data points represent individual participants.

The mean proportion of percepts that matched structural expectations in test trials (hereafter: action-consistent percepts) was submitted to a directional Bayesian one-sample t-test against chance (i.e., 0.50) in all three conditions (JASP version 0.9.1, default Cauchy prior width = 0.707). The proportion of action-consistent percepts exceeded chance level in the baseline (*M* = 0.54, *SD* = 0.05, BF_+0_ = 87.94, Cohen’s *d* = 0.92), and compatible conditions (*M = *0.53, *SD* = 0.05, BF_+0_ = 24.70, Cohen’s *d* = 0.70), but did not deviate from chance in the incompatible condition (*M* = 0.50, *SD* = 0.06, BF_−0_ = 0.16, Cohen’s *d* = 0.08). Subsequent two-sided pair-wise comparisons with the baseline condition showed that the proportion of action-consistent percepts was smaller in the incompatible condition than in the baseline condition (BF_10_ = 3.00, Cohen’s *d* = 0.74). In contrast, the evidence for the *absence* of a difference between the baseline and compatible condition was about three times as large as the evidence for a difference in the proportion of action-consistent percepts (BF_10_ = 0.36, Cohen’s *d* = 0.19). However, note that the evidence for both of these between-group differences is relatively weak. No adjustments for multiple testing were applied, as type I and type II errors do not apply to Bayesian tests^18,19^. The proportion of action-consistent percepts for all conditions is depicted in Fig. 1.

We refrained from statistically testing assumptions of normality and homogeneity of variance as these tests would have little power as a result of the relatively small sample sizes. Importantly, however, visual inspection of the data (see Fig. 1) suggests the absence of any gross assumption violations.

#### Effect over time

In order to examine whether the observed biases would change over time, the proportion of action-consistent percepts was compared between the first and the second half of the test phase in all conditions (i.e., compatible learning, incompatible learning, and baseline). Directed paired sampled t-tests were used for the compatible and incompatible conditions as we expected potential extinction effects of the learned contingencies. Specifically, we expected the proportion of action-consistent percepts to reduce in the compatible condition and to increase in the incompatible condition (i.e., moving towards the baseline). In the baseline condition, a non-directional paired sampled Bayesian *t*-test was used. Unexpectedly, time effects were observed in none of the conditions (Baseline: BF_10_ = 0.38; Compatible: BF_+0_ = 0.20; Incompatible: BF_−0_ = 0.11, see Table 1).

**Table 1:** Descriptives of action-consistent percepts over time in Study 1.

	Time 1 (first half)		Time 2 (second half)		Cohen’s d
	Mean	SD	Mean	SD	
Baseline	0.53	0.06	0.56	0.08	−0.24
Compatible	0.53	0.08	0.54	0.08	−0.04
Incompatible	0.52	0.07	0.50	0.08	0.22

Cohen’s *d* represents the effect size for time 1 ≠ time 2 for the baseline condition, time 1 > time 2 for the compatible condition and time 1 < time 2 for the incompatible condition.

#### Induction trials

The propensity of participants to perceive action-outcomes in line with their expectations was also evaluated in the induction trials. In contrast to test trials, induction trials contained objective switches in the rotation direction of the sphere, and as such the perceived switches on these trials could be objectively (in)correct. Performance (in terms of the proportion of accurate trials) was near perfect for both learning conditions, and did not depend on whether the initial sphere motion was compatible (compatible learning condition: *M*_*acc*_ = 0.98, *SD*_*acc*_ = 0.05, incompatible learning condition: *M*_*acc*_ = 0.97, *SD*_*acc*_ = 0.07) or incompatible (compatible learning condition: *MD*_*acc*_ = 0.98, *SD*_*acc*_ = 0.06, incompatible learning condition: *M*_*acc*_ = 0.97, *SD*_*acc*_ = 0.03) with the preceding rotary action. Note that these accuracy levels are calculated after excluding error trials (see “Data exclusion” section) and are based on a different number of trials for the compatible and incompatible condition (see “Procedure and design section”). The near-ceiling performance demonstrates that participants understood the instructions and paid attention to the task.

### Study 2

#### Perceptual selection

**Figure 2:**
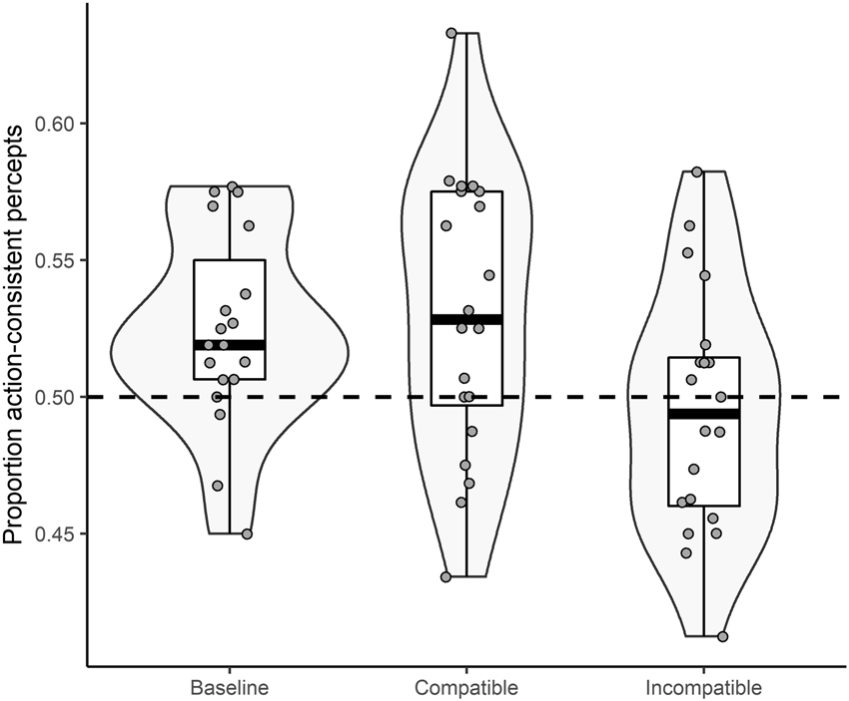
Proportion of action-consistent percepts as a function of experimental condition in Study 2. Lower and upper box limits represent the 25^th^ and 75^th^ percentile respectively. Whiskers extend to the most extreme values that fall within 1.5 times the interquartile distance from the hinges of the box. Central lines represent the median. Data points represent individual participants.

Our expectations for Study 2 followed from the observed findings of Study 1. We expected participants to be more likely than chance to perceive the bistable stimulus in line with structural (i.e., action-consistent) expectations in the test trials of the baseline and compatible condition. In addition, we expected the proportion of action-consistent percepts in both these conditions to be larger than in the incompatible condition. To test whether we could replicate this specific pattern of results, we formulated the following informative hypothesis (H-inf): μ_baseline_ > 0.5, μ_compatible_ > 0.5, μ_baseline_ > μ_incompatible_, μ_compatible_ > μ_incompatible_. The evidence for this hypothesis was compared against its complement H-c (i.e., not H-inf). This analysis yielded strong evidence for the expected pattern (BF_inf,c_ = 208.62), suggesting the overall observed pattern of Study 1 was replicated (*M*_*base*_ = 0.52, *SD*_*base*_ = 0.04; *M*_*comp*_ = 0.53, *SD*_*comp*_ = 0.05; *M*_*incomp*_ = 0.49, *SD*_*incomp*_ = 0.04; see Fig. 2).

To further examine this pattern of results, several pre-registered follow-up analyses were executed. Firstly, to further evaluate the specific evidence for the presence of a structural expectation, we compared the informative hypothesis (H-inf) μ_baseline_ > 0.5, against the competing hypothesis (H-c) μ_baseline_ = 0.5. This analysis yielded evidence for a proportion of action-consistent percepts that exceeds chance level (BF_inf,c_ = 4.97, Cohen’s *d* = 0.69). In addition, we observed evidence for the *absence* of a difference between the baseline and compatible condition (H-inf: μ_baseline_ = μ_compatible_, tested against H-c: μ_baseline_ < μ_compatible;_ BF_inf,c_ = 5.35, Cohen’s *d* = 0.13 (reflecting the effect size for μ_baseline_ < μ_compatible_)). The evidence for a difference between the baseline and the incompatible condition went in the same direction as in Study 1, but was not conclusive (H-inf: μ_baseline_ > μ_incompatible_, tested against H-c: μ_baseline_ = μ_incompatible_; BF_inf,c_ = 2.49, Cohen’s *d* = 0.75). Finally, similar to Study 1, the incompatible condition was not different from chance (H-inf: μ_incompatible_ = 0.5, tested against H-c: μ_incompatible_ ≠ 0.5. BF_inf,c_ = 6.61, Cohen’s *d* = 0.13 (reflecting the effect size for μ_incompatible_ ≠ 0.5)). No explicit assumption tests nor adjustments for multiple comparisons were performed.

#### Effect over time

No evidence for a difference in the proportion of action-consistent percepts as a function of time was observed for any of the conditions in Study 1. To test this specific pattern of results we compared the evidence for the informative hypothesis H-inf: μ_baseline1_ = μ_baseline2_, μ_compatible1_ = μ_compatible2_, μ_incompatible1_ = μ_incompatible2_, with the competing hypothesis H-c: μ_baseline1_, μ_baseline2_, μ_compatible1_, μ_compatible2_, μ_incompatible1_, μ_incompatible2_. Coinciding with the results of Study 1, we found evidence for the *absence* of time effect (BF_inf,c_ = 4.26; see also Table 2).

**Table 2:** Descriptives of action-consistent effects over time in Study 2.

	Time 1 (first half)		Time 2 (second half)		Cohen’s d (time1 ≠ time2)
	Mean	SD	Mean	SD	
Baseline	0.52	0.04	0.53	0.05	0.02
Compatible	0.53	0.08	0.53	0.09	0.05
Incompatible	0.48	0.08	0.51	0.05	0.25

#### Induction trials

Similar to Study 1, accuracy levels were near ceiling in both the compatible and incompatible induction blocks, and did not depend on whether the initial sphere motion was compatible (compatible learning condition: *M*_*acc*_ = 0.98, *SD*_*acc*_ = 0.04, incompatible learning condition: *M*_*acc*_ = 0.96, *SD*_*acc*_ = 0.09) or incompatible with the preceding action (compatible learning condition: *M*_*acc*_ = 0.97, *SD*_*acc*_ = 0.09, incompatible learning condition: *M*_*acc*_ = 0.97, *SD*_*acc*_ = 0.05).

## Discussion

Across two studies we observed that both structural and contextual expectations affect the perception of action-outcomes. Interestingly, these effects did not seem to operate in a simple additive manner. That is, the bias induced by already existing expectations was not further enhanced by compatible contextual expectations. In addition, incompatible learning abolished, but did not reverse, existing structural biases. All the observed effects were stable over time. The implications of these findings are discussed below.

The modulation of perception by action-based predictions has been examined extensively and is generally attributed to internal forward models implicated in motor control^20,21^. Specifically, when one prepares an action, forward models are thought to use a copy of the motor command to predict the sensory consequences of the action, which in turn influences perceptual processing^4,5^. These forward models are generally assumed to be highly adaptable in order to allow for efficient behavior in a constantly changing environment. Indeed, research on motor adaptation suggests that people can concurrently acquire multiple internal models, and flexibly switch between these models based on task demands (e.g.^22^). To the best of our knowledge, however, it thus far remains unknown how action-based expectations that developed across different timescales are combined.

In the present studies we show that pre-existing, overlearned expectations are quickly abolished by short exposure to disconfirming evidence. Similar updating effects have been observed in research examining the opposite side of the action-perception link. Specifically, the generally automatic (structural) tendency to imitate an observed action^23^, can be abolished through learning^24^, or even be replaced by a tendency to perform complementary actions as a result of instructed task rules^25,26^. Together these findings point towards a strong top-down influence of contextual information on how the motor and perceptual system interact. However, the mechanism that underlies such contextual influences is still a matter of debate. According to the MOSAIC model, people can switch between multiple, motor-specific, internal models that exist alongside each other^27^. In contrast, more recent accounts suggest that there is only one, context-sensitive, predictive model that is not specific to motor predictions, but rather involves predictions about sensory events in general^28,29^. The observed flexibility in our current studies can be explained by both models, and future research is necessary to elucidate the exact processes via which these updating effects come about.

The present results are also in accordance with previous work on the updating of structural, action-based, predictions^14^. Similar to the present results, this study demonstrated that the pre-existing bias to perceive ambiguous motion in the same direction as one’s action can be altered by learning. Unlike the present findings, however, the structural bias was enhanced by compatible learning and reversed by incompatible learning. One potential explanation for these diverging results, is that no consistent effect of structural expectations was observed in the previous study. In the absence of such a (strong) structural bias, perception might be more amendable by recently acquired contextual expectations. In contrast, the clear structural bias observed in the present study could be at the limits of the malleability of perception, restricting further enhancement by compatible learning. Likewise, this strong structural bias might have been relatively difficult to break down by incompatible learning, explaining why incompatible contextual learning was sufficient for abolishing but not for reversing the extant structural bias.

This line of reasoning is corroborated by another recent study examining the joint influence of long-term and short-term expectations on action-outcome monitoring^30^. In this study participants performed a simple ideomotor task in which one of two keys could be used to add a puzzle piece to the top or bottom of another piece presented in the center of a computer screen. Actions could either result in puzzle pieces appearing at the intended (spatially compatible) location or at an incompatible location. Incompatible outcomes were shown to reduce response speed in a subsequently performed, unrelated task. Importantly, this interference effect was modulated by the type of expectations that participants could rely on. Specifically, in some blocks, outcomes were produced at random, which meant that monitoring could only be affected by the pre-existing (long-term) expectation that actions generally lead to compatible outcomes. In contrast, in other blocks participants were exposed to probabilistic action-outcome contingencies that were either compatible (high-compatible blocks) or incompatible (high-incompatible blocks) with this long-term bias. Whilst a strong interference effect was observed in both random and high-compatible blocks, this effect disappeared in blocks in which the majority of trials was incompatible. In other words, much like the present study, incompatible short-term expectations led to the disappearance, but not a reversal, of a pre-existing (structural) processing bias.

Contrary to our expectations, the influence of contextual learning on the perception of action-outcomes did not diminish over time. The absence of such an extinction effect might suggest that the observed influences of learning did not emerge as a result of repeated exposure to contingencies. Instead these effects might have resulted from an all-or-none adherence to the instructed task-set (i.e., the sphere will rotate in the same or a different direction as your action) that was expected to remain stable over the course of the experiment. The absence of a time effect has to be interpreted with some caution, though, as the observed evidence in Study 2 was not robust to variations in prior width (see “Supplementary Results” section in the Supplementary Information). Nevertheless, the findings are in line with research showing temporal stability of general instruction effects on bistable perception. For instance, participants who were made to believe that a specific set of glasses would change the apparent rotation direction of a sphere, were biased to perceive a subsequent bistable sphere in the instructed direction - and continued to do so in the absence of any further evidence as long as the glasses were worn^31^. Likewise, research on action-effect learning has shown that instructed action-outcome beliefs are remarkably resistant against disconfirming evidence after one’s expectations have been consolidated by initial evidence^32^. In the present study the transition of the induction to the test phase was very subtle. Similar to the aforementioned work it is therefore likely that participants held on to the beliefs that were formed based on the combination of structural expectations and initial contextual evidence (i.e., my action either does or does not cause the sphere to move in the same direction as my movement) throughout the experiment.

In conclusion, the present studies demonstrate that the perception of action-outcomes is jointly affected by long-term and short-term expectations. Structural motor expectations that are likely to be formed over the duration of one’s lifetime were abolished after short exposure to incompatible action-effect contingencies. Although this malleability of perception appears to be restricted by the strength of the existing bias (i.e., no reversal effect was observed) these findings suggest a remarkable adaptability of even strong and solidified motor expectations. Considering that humans live in a constantly changing world in which perceptual expectations regularly need to be updated (e.g., when reverse parking a car with a trailer, or when required to drive on the opposite side of the road in a foreign country), this flexibility of our predictive system represents a crucial function for efficient behavior.

## Methods

### Study 1

#### Sample size determination

Sample size was determined by means of a pre-specified stopping rule. This rule prescribed termination of data collection as soon as substantial evidence (i.e., a Bayes factor of 6^33^) was reached for the presence or absence of action-consistent effects, after inclusion of a minimum of twenty participants per condition^34,35^. The stopping rule for the compatible condition slightly deviated from the other conditions (see “Sample size determination” section in the Supplementary Information). We defined action-consistent percepts as percepts that are in line with structural expectations (i.e., a clockwise sphere motion following a clockwise rotary action).

#### Participants

A total of seventy-one participants took part in one of three experimental conditions, which were run as sequential experiments. Twenty participants were assigned to the baseline condition (*M*_*age*_ = 21.20, *SD*_*age*_ = 1.67, three left handed, sixteen females), twenty-five participants were assigned to the compatible condition (*M*_*age*_ = 22.52, *SD*_*age*_ = 2.42, two left handed, nineteen females), and twenty-six were assigned to the incompatible condition (*M*_*age*_ = 21.50, *SD*_*age*_ = 1.86, four left handed, twenty-four females). None of the participants took part in more than one condition. Prior to the experiment, a pre-screening for stereoscopic vision was completed (see “Pre-screening Procedure” section in the Supplementary Information). The studies were conducted in accordance with the Declaration of Helsinki and the used paradigm is approved by the ethics board of the Faculty of Social Sciences at Utrecht University. Participants provided informed consent prior to the start of all studies and received money and/or course credit in exchange for participation.

#### Apparatus

Participants were seated in front of a mirror stereoscope consisting of two mirrors at a 45-degree angle, which each reflected one of two opposite linearized 23-inch LCD monitors (Dell UZ2315H; resolution: 1920 × 1080; refresh rate: 60 Hz). This set-up has been described in detail elsewhere^36^. A stabilized head position and constant viewing distance of approximately 82 centimeters was ensured by a chin and forehead rest.

#### Stimuli

Participants were exposed to disambiguated and ambiguous visual structure-from motion spheres. The spheres were adapted from those used in previous research^37^, and consisted of 240 white, square “dots”, half of which moved in a leftward direction, and half of which moved in a rightward direction. The speed of the dots was 45 degrees of visual angle (dva) per second in the center and decreased to zero toward the edges of a circular aperture following a sinusoidal, thus eliciting the percept of a rotating sphere. Each dot had a lifetime of one second after which it was replaced by a new, randomly positioned dot. Initial dots were assigned a random ‘age’ between zero and one seconds to prevent simultaneous replacement of all dots. The rotation direction of the resulting structure-from-motion spheres was intrinsically ambiguous. Dots moving in the same direction are typically interpreted as belonging to a similar depth plane, and depending on whether the leftward dots or the rightward dots appear closer to the observer, the sphere is perceived as rotating leftwards or rightwards, respectively. Stimuli were presented using the Psychophysics Toolbox 3^38,39^ in Matlab R2016b (The Mathworks, Natick, MA).

In this study, both spheres with an ambiguous rotation direction (in test trials), and spheres with a disambiguated rotation direction (in induction trials) were used. Similar spheres were separately presented to the two eyes of the participants, and different horizontal offsets between the eyes (i.e., disparities) were added to the leftward and rightward moving dots. This caused dots moving in one direction to appear closer to the observer than dots moving in the other direction, hence disambiguating the rotation direction of the spheres. Disparities ranged between 0 and 0.04 dva relative to fixation. To create spheres with an ambiguous rotation direction the disparity was set to 0 (i.e., the two eyes’ images were identical).

Intermittent presentation of ambiguous spheres is known to induce perceptual stabilization effects. Specifically, the perceptual interpretation of intermittently presented bistable stimuli is strongly biased towards the previous percept^40^, potentially causing the initial percept to persist across successive trials for more than ten minutes^41^. In order to reduce these influences, the location of both ambiguous and unambiguous spheres varied randomly between one of four quadrants around the fixation point^42^.

#### Procedure and design

Throughout the experiment, participants initiated the rotation of a sphere by rotating a custom-made rotary switch. This switch was designed to induce a strong mapping between action and motion as the rotary movements of participants were parallel to the sphere’s rotation plane.

Participants took part in one of three conditions: the baseline (i.e., no-learning) condition, the compatible learning condition or the incompatible learning condition. The baseline condition was designed to assess the influence of participants’ structural action-outcome expectations on perception (i.e., the tendency to see a clockwise rotating sphere after rotating the switch in a clockwise direction). Accordingly, this condition only comprised of a test phase, in which the rotation direction of the sphere was ambiguous. Additionally, the compatible and incompatible learning conditions were designed to manipulate participants’ contextual action-outcome expectations. In these two conditions, the test phase was preceded by a learning phase (40 trials), in which participants used the rotary switch to set the sphere into motion with an unambiguous rotation direction. Rotary actions were followed by sphere motion directions that were either 80% compatible or 80% incompatible with existing structural expectations. Participants were explicitly informed on the direction of these contingencies. In the subsequent test phase (80 trials), all rotary actions were followed by a sphere with an ambiguous rotation direction.

**Figure 3:**
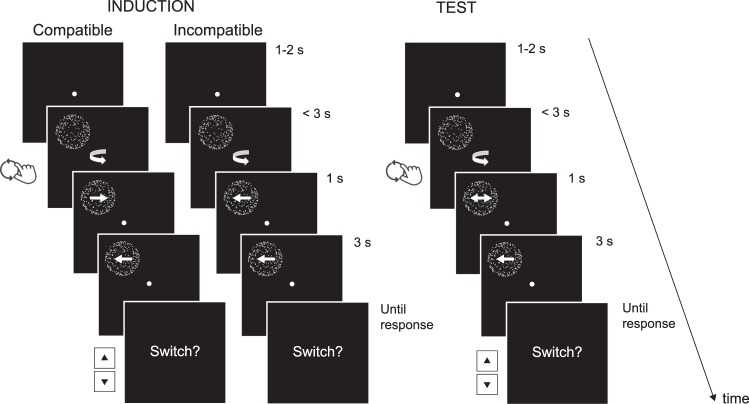
Schematic representation of trial events in the induction phase and the test phase.

The timeline of both induction and test trials is depicted in Fig. 3. Each trial started with the presentation of a fixation point in the center of the screen. After a random duration of 1000 to 2000 milliseconds (with intervals of 250 milliseconds) a stationary sphere appeared. As soon as the sphere appeared on the screen, the fixation point was replaced by an action-cue (a clockwise or counterclockwise arrow), which indicated the direction in which the rotary switch had to be rotated. Participants handled the switch with their dominant hand. If participants rotated the switch within the time limit of three seconds, the stationary sphere started to move. Incorrect rotations were followed by an error message. In order to motivate participants to perform the correct actions, they could earn money for correct rotations and lose money for incorrect rotations. Unbeknownst to the participants, the earned amount was rounded up and all participants were paid the same amount.

In induction trials, the initial rotation direction of the sphere was either compatible or incompatible with the direction in which the rotary switch was moved by the participants. After one second, the sphere either continued to rotate in the same direction for three seconds, or switched and rotated in the opposite direction for three seconds (crucially, only the initial rotation direction was conditional upon the participant’s action). Participants were asked to indicate whether they perceived a switch by pressing the “up” arrow key for “yes” and the “down” arrow key for “no”. In test trials, the rotation direction of the sphere was ambiguous in the first second, and was unambiguous in the last three seconds (50% clockwise, 50% counterclockwise). Registering switch reports (rather than percept reports) allowed us to infer the perceived initial rotation direction, while minimizing demand characteristics (i.e., participant’s tendency to report what they think they should have seen, instead of what they actually perceived).

The task consisted of five blocks of eight induction trials (in the compatible and incompatible learning conditions) and ten blocks of eight test trials (in all three conditions). Each possible combination of the required rotation of the switch (clockwise versus counterclockwise) and the second unambiguous rotation period of the sphere (clockwise versus counterclockwise) was shown twice in each induction and test block in a randomized order. Action-outcome contingency was kept at 80/20 for the compatible condition and at 20/80 for the incompatible condition, across all induction blocks. All four sphere locations were each shown ten times in the induction phase and twenty times in the test phase. The order of the location was randomized, with the restriction that no location could be shown twice in a row. Prior to the experiment participants completed two practice rounds to get familiarized with the switch (8 trials) and to practice with the task (20 trials in the learning conditions and 8 trials in the baseline condition).

### Data exclusion

#### Induction trials

Trials were excluded from analysis when the switch was rotated in the wrong direction (i.e., not in the direction that was indicated by the action-cue; Compatible: *M* = 0.20%, *SD* = 0.69%; Incompatible: *M* = 0.48%, *SD* = 2.00%). On top of that, we also excluded trials in which the switch was incorrectly handled, such as when it was rotated too early (before the presentation of the cue), too late (not within the response time limit), not far enough, too far, back and forth, or more than once; Compatible: *M* = 0.90%, *SD* = 1.22%; Incompatible: *M* = 1.73%, *SD* = 2.09%).

Accuracy levels for the perceived switches in motion direction were calculated over the remaining induction trials. Two participants were excluded from further data analysis for performing at (or below) chance level in the compatible condition. In addition, another participant in this same condition was excluded from analysis for scoring significantly lower than the overall group (z-score = −3.81, calculated after excluding the aforementioned participants). The low accuracy score of these participants suggests that they were not able to distinguish between the different disambiguated rotation directions. One additional participant was excluded in the baseline condition due to insufficient stereoscopic vision (see “Pre-screening procedure” section in the Supplementary Information for details).

#### Test trials

Similar to the induction trials, test trials were excluded when the switch was rotated in the opposite direction as the action-cue (Baseline: *M* = 0.13%, *SD* = 0.39%; Compatible: *M* = 0.06%, *SD* = 0.27%; Incompatible: *M* = 0.72%, *SD* = 1.84%), and when the switch was incorrectly handled (Baseline: *M* = 1.18%, *SD* = 1.79%; Compatible: *M* = 0.97%, *SD* = 2.25%; Incompatible: *M* = 1.01%, *SD* = 2.21%). The reported percentages were calculated after participant exclusion.

### Study 2

To establish the robustness of the observed results, we replicated Study 1 in a pre-registered study, of which the pre-registration form can be accessed at osf.io/bmxwv. The pre-registration comprises an exhaustive description of all planned data pre-processing and analyses, so that no parameters could be adjusted after data collection was completed^43^. Note that this replication attempt also allowed us to confirm whether the observed pattern of results in study 1 was indeed caused by the experimental manipulations, rather than by pre-existing, between-group differences. Study 2 was identical to the previous experiment with a few exceptions. Firstly, the contingency between the participants’ action and the ensuing sphere rotation was now kept at 80% *within* each individual block of the induction phase (rather than across blocks, as in Study 1), resulting in a fully balanced design. Secondly, evidence for the expected pattern of results was tested by means of Bayesian model selection^44^, using the R package Bain^45^. This approach allowed us to compare a model that reflects our predictions for all conditions simultaneously, against a model in which our specific set of predictions are not met (see Results section for details). Bain’s Markov chain Monte Carlo settings are chosen such that the program itself determines whether or not the sampling procedures have converged, and accurate estimates of complexity, fit and Bayes factors have been obtained (for details see:^45–47^).

#### Participants

Sixty-three participants took part in the experiment (*M*_*age*_ = 22.27, *SD*_*age*_ = 2.20, nine left handed, forty-eight females).

### Data exclusion

#### Induction trials

Similar to Study 1, trials were excluded when the switch was rotated in the wrong direction relative to the action-cue (Compatible: *M* = 0.57%, *SD* = 2.67%; Incompatible: No errors). On top of that, trials in which the rotary switch was incorrectly handled were also excluded (Compatible: *M* = 1.59%, *SD* = 1.97%; Incompatible: *M* = 1.67%, *SD* = 2.54%).

In addition, in line with the pre-registered exclusion criteria, three participants (two in the compatible condition and one in the incompatible condition) were replaced before data analysis for showing suboptimal accuracy in perceiving switches in motion direction (<80%) in the induction phase. For practical reasons these participants were not replaced immediately, as described in the pre-registration. Instead, the participants were replaced in a random order after running twenty participants per condition. This resulted in a final sample of twenty participants in each condition. After completing data collection, one additional participant was excluded from the baseline condition for being an outlier according to pre-registered criteria (i.e., the mean proportion of action-consistent percepts exceeded 1.5 times the interquartile range from the 1^st^ quartile for the baseline condition).

#### Test trials

Trials were excluded when participants rotated the switch in the opposite direction of the action-cue (Baseline: *M* = 0.59%, *SD* = 1.21%; Compatible: *M* = 0.38%, *SD* = 1.68%; Incompatible: *M* = 0.38%, *SD* = 1.00%). In addition, trials in which the rotary switch was incorrectly handled were also excluded (Baseline: *M* = 0.72%, *SD* = 1.05%; Compatible: *M* = 1.38%, *SD* = 1.57%; Incompatible: *M* = 1.00%, *SD* = 0.96%). The reported percentages were calculated after participant exclusion.

## Supplementary information

Supplementary Information

## Data Availability

Data to reproduce the reported results can be accessed at https://osf.io/9xq6v/. This includes data to reproduce Figs 1 and 2.

## Code Availability

Code for data pre-processing and analysis can be accessed at https://osf.io/9xq6v/. Matlab code for running the experiment is available upon reasonable request from M.D.
