# Supplementary material for: Perception of action-outcomes is shaped by life-long and contextual expectations
Source: Sci Rep. 2019 Mar 26;9:5225. doi: 10.1038/s41598-019-41090-8 (PMC6435663; doi:10.1038/s41598-019-41090-8)
Supplement: Supplementary file 1 — Supplementary Information [file 41598_2019_41090_MOESM1_ESM.docx]

**Perception of action-outcomes is shaped by life-long and contextual expectations *[Supplementary Information]***

Myrthel Dogge^1*^, Ruud Custers^1^, Surya Gayet^2^, Herbert Hoijtink^3^ and Henk Aarts^1^

**Supplementary Methods**

**Sample Size Determination**

**Study 1.** Sample size was based on a pre-specified stopping rule (see Methods section main document). For the compatible condition (which was run first) this rule was slightly different than for the other two conditions. Specifically, data collection was planned to be terminated as soon as substantial evidence was found for (or against) a learning effect in the *first half* of the test trials. This rule was based on the expected extinction of contextual learning over time. Data collection was stopped despite not meeting this pre-specified criterion for several reasons. First, after twenty-five participants the Bayes factor still failed to exceed one of the evidence thresholds (i.e., a BF of 6 or 1/6). Second, the proportion of action-consistent percepts was constant over time, and substantial evidence for a prediction effect was observed across all trials. Based on these observations, the stopping rule was changed for subsequent experiments. Note that this posteriori choice is unlikely to have affected the presented results, which is further evidenced by the replication in Study 2.

**Study 2.** The stopping rule for Study 2 has been described in detail in the pre-registration document (osf.io/bmxwv).

**Pre-screening Procedure**

**Study 1.** In order to check whether participants had normal stereoscopic vision, they took part in a simple disparity task. In this task, participants were exposed to a 3D black annulus in the center of the screen. Stereoscopic depth information was provided by adding a horizontal offset to the annulus that was presented to each eye, relative to fixation (i.e., binocular disparity). The amount of horizontal offset was varied by means of two adaptive, stochastic approximation staircases^1^ that converged to an accuracy level of 80 percent after 30 trials. On each trial, participants were asked to report the depth plane in which the annulus was perceived. Correct and incorrect responses resulted in a decrease and an increase of the offset on the next trial, respectively. The minimum offset that resulted in a stable depth percept was determined by averaging the last eleven offsets for both staircases. Only participants for whom this minimum offset was equal to or smaller than 0.04 dva were invited to participate in the actual experiment. We initially used a cut-off of 0.07 dva for inclusion. However, during data collection in a parallel project it became clear that a stricter threshold of 0.04 dva is preferable to ensure that participants reliably observe unambiguous sphere directions. All but one of the already included participants met this criterion. This participant was excluded from analyses as described in the result section of Study 1.

**Study 2**. In Study 2 an adjusted screening procedure was applied. The pre-screening consisted of two short computer tasks checking for visual acuity and stereoscopic vision. The visual acuity task was a computerized variant of the Landolt-C task, in which the letter “C” was presented in the center of the screen at an angle of 0, 90,180 or 270 degrees. Participants were tasked to indicate the position of the gap by means of the arrow keys, which caused the letter to change in size on the subsequent trial. The size of the letter was determined via an adaptive staircase procedure that converged to an accuracy level of 80 percent after 40 trials. The minimum gap that was perceived correctly on at least 80% of the trials was calculated by averaging the last ten gap widths. Participants who were not able to distinguish a gap equal to or smaller than 0.04 dva were not invited to participate in the main experiment.

To test for stereoscopic vision, participants were exposed to the actual stimuli used in the task (instead of to an annulus as was the case for Study 1). Accordingly, they viewed twenty spheres with a disambiguated motion direction. Participants were asked to report the rotation direction of the sphere by means of the arrow keys (e.g., pressing the left key when the front of the sphere was moving in a leftward direction). Participants who were not able to correctly indicate the correct rotation direction on at least 80% of the trials, were not invited to participate in the main experiment.

**Supplementary Results**

**Sensitivity Analyses**

**Study 1.** In order to check the robustness of the drawn conclusions under changing prior models, robustness regions (RR) were calculated. These regions reflect the range of cauchy prior widths under which the same scientific inferences can be drawn (we used BF ≥ 3 in favor of the reported conclusion). The regions for all tests are depicted in Supplementary Table 1. Note that JASP does not allow the entry of a cauchy prior width greater than 2. Accordingly, prior widths exceeding this threshold are not evaluated.

Supplementary Table 1.

*Robustness ranges for Study 1*

|  | **Main effect** | **Time effect** |
| --- | --- | --- |
| Baseline condition | RR[0.01, 2] | RR[0.85, 2] |
| Compatible condition | RR[0.02, 2] | RR[0.37, 2] |
| Incompatible condition | RR[0.29, 2] | RR[0.18, 2] |
| Baseline vs. Compatible | RR[0.78, 2] | NA |
| Baseline vs. Incompatible | RR[0.39,0.70] | NA |

**Study 2**. In contrast to JASP, the variance of the prior distribution that is used in Bain is estimated based on a fraction of the data. Accordingly, a different method of determining the robustness of drawn inferences is appropriate. Specifically, a sensitivity analysis can be executed in which the fraction that is used to estimate the prior is varied (for more information visit: https://informative-hypotheses.sites.uu.nl/software/bain/^2^). Considering that the variance of the prior only affects hypotheses that include equality constraints^3^, sensitivity analyses will not be reported for tests restricted to inequality constraints. The sensitivity analyses were not pre-registered. Supplementary Figure 1 depicts the sensitivity analyses for the follow-up tests that were described in detail in the main document. In addition, we also conducted a sensitivity analysis for the time effect across the three conditions. The evidence for the absence of a time effect becomes inconclusive as the prior width decreases (BF_inf,c =_ 1.51 and BF_inf,c_ = 0.82 for a b_g_ of two and three respectively).


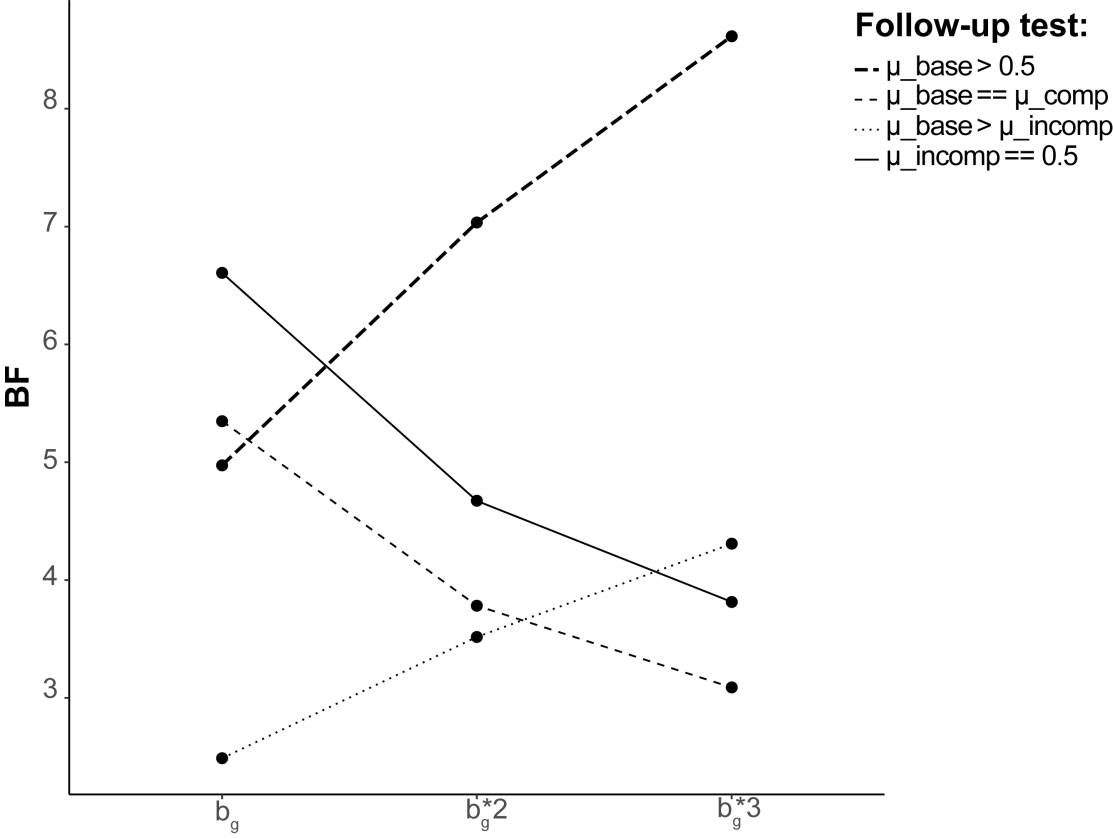


*Supplementary Figure 1*. Sensitivity analyses for the follow-up tests of Study 2. The Bayes factor is plotted as a function of the fraction of the data that was used to estimate the prior variance (b_g_). This variance decreases as the fraction increases.

**References**

1. Kesten, H. Accelerated stochastic approximation. *Ann. Math. Stat.* **29,** 41–59 (1958).

2. Hoijtink, H., Mulder, J., van Lissa, C. & Gu, X. Tutorial : Testing hypotheses using the Bayes factor. *Psychol. Methods.* (2018).

3. Gu, X., Mulder, J. & Hoijtink, H. Approximated adjusted fractional Bayes factors: A general method for testing informative hypotheses. *Br. J. Math. Stat. Psychol.* **71,** 229–261 (2018).
